# The opioid crisis is driving mortality among under-served people living with HIV in British Columbia, Canada

**DOI:** 10.1186/s12889-021-10714-y

**Published:** 2021-04-08

**Authors:** Kate A. Salters, Stephanie Parent, Valerie Nicholson, Lu Wang, Paul Sereda, Tatiana E. Pakhomova, Mia Kibel, William Chau, Kalysha Closson, Surita Parashar, Rolando Barrios, Julio S. G. Montaner, Robert S. Hogg

**Affiliations:** 1 British Columbia Centre for Excellence in HIV/AIDS, St. Paul’s Hospital, Vancouver, Canada; 2 Faculty of Health Sciences, Simon Fraser University, Burnaby, BC V5A 1S6 Canada; 3 Dalla Lana School of Public Health, University of Toronto, Toronto, Canada; 4 School of Population and Public Health University of British Columbia, Vancouver, Canada; 5 Vancouver Coastal Health, Vancouver, Canada; 6 Faculty of Medicine, University of British Columbia, Vancouver, BC Canada

**Keywords:** Mortality, HIV/AIDS, Overdose, Chronic disease, Antiretroviral therapy

## Abstract

**Introduction:**

Universal provision of effective antiretroviral medication has been essential to reduce mortality, increase longevity, and reduce onward transmission of HIV. This study aims to illuminate persistent threats to the health and longevity of under-served PLWH in British Columbia (BC), Canada.

**Methods:**

Between 2007 and 2010, 1000 PLWH across BC were enrolled in the Longitudinal Investigation into Supportive and Ancillary health services (LISA) study and completed a cross-sectional survey on their HIV-care experiences and healthcare engagement. The sample generally reflects an under-served population of PLWH. A linkage to the provincial Vital Statistics registry is used in this analysis in order to examine overall mortality and cause-specific mortality trends; probability of death was modeled using logistic regression for participants with ongoing clinical monitoring (*n* = 910).

**Results:**

By June 2017, 208 (20.8%) participants had died. The majority of deaths 57 (27.4%) were attributed to drug-related complications or overdoses, 39 (18.8%) were attributed to HIV-related complications, and 36 (17.3%) to non-AIDS-defining malignancies. We observed elevated odds of death among PLWH who smoked tobacco (aOR: 2.11, 95% CI: 1.38, 3.23), were older (aOR: 1.06 per one-year increase, 95% CI: 1.04, 1.08), indicated heavy alcohol consumption (aOR: 1.57, 95% CI: 1.11, 2.22), and reported unstable housing (aOR: 1.96, 95% CI: 1.37, 2.80); while higher CD4 cell count was protective (aOR: 0.87 per 100-unit increase, 95% CI: 0.79, 0.94) as was male gender), though non-significant (aOR: 0.73, 95% CI: 0.49, 1.07).

**Conclusions:**

Overdose is - the leading cause of mortality among a cohort of under-served PLWH in BC, Canada. Public health efforts to end the HIV epidemic and support the health and well-being of PLWH are being thwarted by persistent health inequities and the enormous and persistent risks facing people who use drugs. Integrated low-barrier primary care is essential for supporting under-served PLWH, and safe drug supply is needed to support PLWH who use drugs.

## Background

A decline in new HIV infections globally and increases in uptake of antiretroviral therapy (ART) globally demonstrates the effectiveness and need for ongoing public health, medical, and community efforts to combat HIV/AIDS [1]. In British Columbia (BC), Canada, evidence-based public health strategies have focused on expanding access to HIV care and treatment for all people living with HIV (PLWH), including universal access to publicly-funded modern antiretroviral therapy (ART) [2]. These strategic efforts, coupled with therapeutic advances in HIV treatment and developments in supportive HIV-care, have contributed to a dramatic reduction in mortality among PLWH in the province [3–5]. Consequently, HIV is now often framed as a manageable chronic illness [6] for many PLWH, as demonstrated by increasing life expectancies in BC [7, 8]. On World AIDS Day (December 1) 2019, the provincial health minister, Hon. Adrian Dix, along with Dr. Julio Montaner, Director of the BC Centre for Excellence in HIV/AIDS (BC-CfE), announced the province has achieved record low numbers of new HIV cases, [9] – a 73% reduction since 1996, indicating a significant shift in the epidemic [10].

While modern Treatment as Prevention efforts help to directly address the HIV epidemic, complex social issues remain primary drivers of health inequities between PLWH and the general population. PLWH experience disproportionate rates of homelessness [11], violence [12], and mental illness [13–15] and they also experience elevated risks for comorbidities such as non-AIDS-defining malignancies (NADM), cardiovascular disease, and substance-use complications [16–20]. This is compounded by the fact that within the last decade, unintentional overdoses and drug-related deaths resulting from the contamination of the drug supply with synthetic opioids, have increased significantly [21]. In response to the ongoing opioid crisis, the BC Ministry of Health declared a public health emergency in April 2016 [22]. The intersecting public health crises of HIV and the opioid crisis should be a priority in order to fully support the health and well-being of PLWH.

Therefore, it is necessary to look at current mortality trends among PLWH impacted by socio-structural inequities who remain under-served by the health system, in a setting where HIV-care is provided free-of-charge. This analysis will help to inform our public health approaches and reorganize our efforts to provide comprehensive and responsive care for all PLWH in the modern era of Treatment as Prevention that presents new and evolving priorities for health and well-being.

## Methods

The analytic sample for this analysis was drawn from the Longitudinal Investigations into Supportive and Ancillary health services (LISA) study. The LISA study was established by researchers, community organizations and members, as well as clinicians in BC in order to evaluate the impact of health care engagement and social determinants of health on PLWH who have accessed ART in BC. Cross-sectional LISA survey data are linked with ongoing longitudinal clinical data from the Drug Treatment Program (DTP) registry at the BC-CfE, which provides real-time clinical monitoring including CD4 cell count, viral load monitoring, resistance testing, and other measures of therapeutic engagement. In BC, ART is distributed free-of-charge through the DTP at the BC-CfE to all PLWH residing in the province [23]. The DTP registry was developed in 1992 and includes data on all PLWH with a viral load and/or receipt of ART.

Between July 2007 and January 2010, the estimated 9514 PLWH enrolled in the DTP registry were eligible to participate in the LISA survey and were invited through study information letters distributed by physicians providing HIV care, posters at pharmacies where patients refilled ART prescriptions, notices posted at HIV/AIDS clinics and service organizations across the province, as well as through word-of-mouth. Recruitment remained open until a sample size of 1000 was satisfied; a target sample size calculated to power specific sub-analyses of interest. A result of convenience and snowball sampling as well as the community-based settings where recruitment often took place, the LISA cohort is not representative of the entire DTP registry and is instead more appropriately represented by under-served populations often experiencing economic and social marginalization including women, those with hepatitis C (HCV) co-infection, and people who use drugs. Eligibility for the study included being able to provide consent into the study, being able to write and speak in English, being over 18 years of age at entry into the study, and having been diagnosed with HIV (as confirmed through the DTP registry).

A cross-sectional survey, collecting information on demographics, health service utilization, and quality of life, was administered to LISA study participants in-person by trained interviewers. A community advisory board and team of researchers developed, reviewed, and piloted the survey questionnaire, which took which took approximately 60 min to complete. Participants were provided with a $20 CAD honorarium for their time and contributions. The LISA study and the DTP have both been previously described at length [24].

### Outcome variable

For this analysis, the outcome variable of interest was death, as identified by the provincial Vital Statistics Agency [25]. These data, including primary cause of death, were made available to the DTP up to June 30th, 2017. Causes of death were further categorized by the lead author in consultation with physician co-authors into the categories represented in Table 1, adapted from categories provided by Statistics Canada by ICD codes. Number of deaths were provided overall and by gender.

### Co-variates

Demographic and socioeconomic variables collected in the LISA cross-sectional survey included self-identified gender (men was inclusive of cis-gender men and trans men while women was inclusive of trans women and cis-gender women), age at time of survey (median, interquartile range [IQR]), self-identified ethnicity (White, Asian, Indigenous, other), current tobacco use (yes or no), excessive alcohol use (CAGE scale), current injection drug use (IDU), perceived stable housing (yes vs. no), education (less than completion of high school diploma vs. completed or more), lifetime self-reported diagnosis of a mental health disorder by a physician (yes vs. no), and current location (rural vs. urban). Lastly, food security was measured based on a modified version of the Radimer/Cornell Questionnaire, and food insecurity was defined as having at least one positive answer to any of the 13 questions [26, 27]. CD4 cell count at baseline was determined by looking at the closest CD4 cell count in the DTP within 3 months of the survey date [referred to as baseline]. We imputed 97 cases of missing CD4 cell count at the time of survey with the mean value of 397 cells/mm^3^.

#### Statistical analyses

Baseline categorical variables were compared in a bivariate analysis using the Chi-square tests or Fisher’s exact test, and continuous variables were compared using the Wilcoxon rank-sum test. Variables with a significant *p*-value (≤0.05) in the bivariate analysis were considered potential factors associated with all-cause mortality. A backward stepwise technique, minimizing the Akaike Information Criterion (AIC), was used in the selection of covariates [28]. We used logistic regression analysis to model the probability of all-cause mortality. Age-standardized mortality rates were calculated using the method outlined by Statistics Canada [29]. We calculated mortality rate comparisons and Fisher’s exact 95% confidence interval (CI) and provided mortality rate ratio with 95% CI. All analyses were performed using SAS software, version 9.4 service pack 3 (SAS Institute, Cary, North Carolina, Version 9).

## Results

Of the 1000 study participants enrolled between 2007 and 2010, 208 (20.8%) had died by June 302,017, as identified by linkage with the Vital Statistics Agency registry database. The primary causes of death (overall and by gender) are categorized in Table 1 below. The most frequent cause of death overall was due to overdose (27.4%), followed by HIV-related complications (18.8%), non-AIDS-related malignancies (17.3%), and respiratory disease or Chronic Obstructive Pulmonary Disease (COPD) (8.2%). Notably, the proportion of over-dose related deaths were higher among women (32.8%) compared to men (25.2%) in our study as were HIV-related deaths (19.7% vs 18.4%).

**Table 1:** Primary cause of death among LISA participants as of June 30, 2017

Primary cause of death	Overall *n* = 208 n (%)	Women *n* = 61 n (%)	Men *n* = 147 n (%)
Drug-use/Overdose	57 (27.4%)	20 (32.8%)	37 (25.2%)
HIV-related	39 (18.8%)	12 (19.7%)	27 (18.4%)
Non-AIDS-related malignancy	36 (17.3%)	7 (11.5%)	29 (19.7%)
Respiratory disease/COPD	17 (8.2%)	9 (14.8%)	8 (5.4%)
Unknown cause	12 (5.8%)	4 (6.6%)	8 (5.4%)
Liver disease	10 (4.8%)	2 (3.3%)	8 (5.4%)
Other	10 (4.3%)	2 (3.3%)	8 (5.4%)
Alcohol-use related	9 (4.3%)	3 (4.9%)	6 (4.1%)
Heart disease	8 (3.8%)	1 (1.6%)	7 (4.8%)
Brain injury/Stroke	5 (2.4%)	0	5 (3.4%)
Injury (intentional and unintentional)	5 (2.4%)	1 (1.6%)	4 (2.7%)

After restricting our sample to participants who had clinical follow-up data in the DTP, and were ART-naïve when entering the DTP registry, a sample of 910 participants remained. Among those 910 PLWH, 194 (21.3%) had died by June 30, 2017 (see Table 2**)**. Those who had passed away were disproportionately represented by PLWH who were (all *p* < 0.01) older (median = 47, Q1-Q3 = 41–53), less likely to identify as White/Caucasian (60.3% vs 66.2%) had lower CD4 cell counts at ART initiation (270 cells/mm^3^ vs. 370 cells/mm^3^), were less likely to be virally suppressed prior to death/end of follow-up (65.5% vs 89.0%). had higher CAGE scores (63.4% vs. 47.7%), and reported currently smoking tobacco (79.8% vs 62.0%). PLWH who passed away in our study were also more likely to be currently using drugs (33.7% vs. 19.2%,), report unstable housing (47.9% vs. 27.4%), and report food insecurity (76.2% vs. 63.9%), compared to PLWH who were alive at the end of follow-up.

**Table 2:** LISA study participants mortality outcomes as of June 30, 2017 (*n* = 910)

Variable	Alive (***n*** = 716)	Died (***n*** = 194)	***p***-value
**Age, at survey**			
Median (Q1, Q3)	45 (39, 50)	47 (41, 53)	0.001*
**Calendar year of ART initiation**			0.149
< 1996	29 (4.1%)	< 5^a^	
1996–2004	419 (58.5%)	124 (63.9%)	
> 2004	268 (37.4%)	65–70^a^	
**Gender**			
Women	197 (27.5%)	60 (30.9%)	0.349
Men	519 (72.5%)	134 (69.1%)	
**Ethnicity**			
White/Caucasian	474 (66.2%)	117 (60.3%)	0.009*
Asian	20 (2.8%)	< 5^a^	
Indigenous	180 (25.2%)	69 (35.6%)	
Other	42 (5.9%)	< 5^a^	
**Current tobacco use**			
Yes	442 (62.0%)	154 (79.8%)	< 0.001*
No	271 (38.0%)	39 (20.2%)	
**Current excessive alcohol use (CAGE)**			< 0.001*
Yes	338 (47.7%)	121 (63.4%)	
No	370 (52.3%)	70 (36.6%)	
**Current injection drug use**			< 0.001*
Yes	137 (19.2%)	65 (33.7%)	
No	576 (80.8%)	128 (66.3%)	
**Stable housing**			
Yes	519 (72.6%)	101 (52.1%)	< 0.001*
No	196 (27.4%)	93 (47.9%)	
**Food security**			
Yes	258 (36.1%)	46 (23.8%)	0.001*
No	456 (63.9%)	147 (76.2%)	
**Location of residence**			
Rural	20 (2.8%)	< 5^a^	
Urban/Suburban	686 (97.2%)	185–195	0.443
**CD4 cell count at ART initiation**			
cells/mm^3^	370 (240, 540)	270 (180, 450)	< 0.001*

Median (Q1, Q3) or n (%) presented

*indicates statistical significance at *p* ≤ 0.05

^a^ For confidentiality and disclosure control measures, exact counts for cells with values *n* < 5 were suppressed. In accordance with strategies employed by Lofters et al. [30], if only one cell was *n* < 5 then one additional category is masked by providing a range (with no percentages available). Disclosure controls are only applied for descriptive tabulations and exact counts were used for analyses

In the multivariable logistic regression model presented in Table 3, we found higher odds of death among those who were older (aOR = 1.06 per 1 year increase, 95% CI: 1.04, 1.08), currently using tobacco (aOR = 2.11, 95% CI: 1.38, 3.23), indicated excessive alcohol use (aOR:1.57, 95% CI: 1.11, 2.22), and reporting unstable housing (aOR: 1.96, 95% CI: 1.37, 2.80), while a higher CD4 cell count at baseline was protective against mortality (aOR = 0.87 per 100-unit increase, 95% CI: 0.79, 0.94) as was male gender (vs women-identified PLWH) (aOR = 0.73, 95% CI: 0.49, 1.07), though statistically non-significant.

**Table 3:** Logistic regression of all-cause mortality among LISA participants (*n* = 893)

Variable	Adjusted Odds Ratio (aOR)	95% Confidence Interval
**Age at baseline**		
per one-year increase	1.06	1.04, 1.08*
**Gender (male)**	0.73	0.49, 1.07
**Current tobacco use**	2.11	1.38, 3.23*
**Indication of excessive alcohol use (CAGE)**	1.57	1.11, 2.22*
**Unstable housing**	1.96	1.37, 2.80*
**CD4 cell count (at baseline)**		
cells/mm^3^ per 100-unit increase	0.87	0.79, 0.94*

*indicates statistical significance at *p* ≤ 0.05

**Fig. 1:**
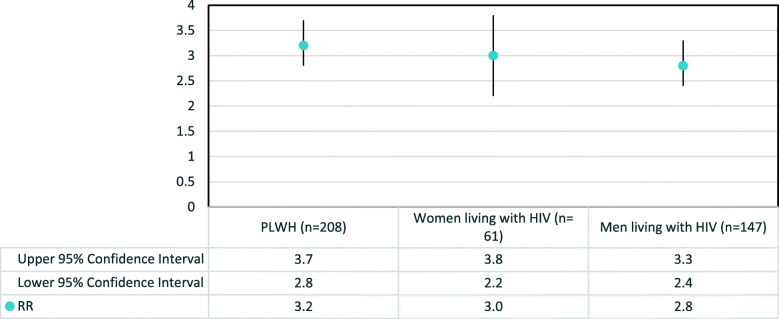
Age-adjusted direct standardized overall mortality rate ratios (RR) with 95% CI compared to 2016 BC general population

We also compared the age-adjusted standardized mortality rates observed in LISA to the 2016 population mortality rates in BC. The age-adjusted standardized mortality rate for PLWH in our study was 25.9 deaths per 1000 PY compared to 8.6 per 1000 PY in the general population [31]. We found an overall elevated rate ratio (RR) of death of 3.22 (95% CI: 2.78, 3.66) among PLWH in our study compared to the general population. After stratifying by gender, the risk of death remained elevated for both men (RR = 2.8) and women living with HIV (RR = 3.0) in our study, compared to the population in BC **(**Fig. 1**).**

## Discussion

This study demonstrates that the universal provision of HIV care and treatment alone does not adequately address the underlying structural and social inequities driving mortality among under-served PLWH. Our study found a more than three-fold increased risk of mortality among under-served PLWH in a setting where ART is publicly-funded. The factors associated with mortality in our cohort, including heavy alcohol use and unstable housing, have been corroborated in other studies, and highlight the persistent social inequities facing many PLWH [32, 33]. This work underscores the need for comprehensive, preventative health care and the need to support PLWH with established risk factors including smoking, heavy alcohol use, and food insecurity.

In particular, this study demonstrates how the current opioid crisis undermines ongoing public health efforts to support the health and well-being of PLWH in BC. In BC, deaths from illicit drug use have been increasing dramatically since 2010, a trend driven by a poisoned market and prohibition policies. In spite of the significant scale-up of harm-reduction strategies and increased access to opioid agonist therapies [34, 35] reported overdose and drug-related deaths across the province were at 1450 at the end of 2017, a significant rise from the 211 drug-related deaths recorded in all of BC in 2010 [36]. Unfortunately, BC is not unique in this, as the rate of overdoses have increased by 500% since 1999 in the United States as well [37]. It is worth noting that the data we have captured in this analysis are representative of only the first 14 months of the provincial public health emergency and, as such, we expect the number of deaths attributed to overdose will continue to rise and disproportionately impact PLWH [19]. Indeed, it is likely that many of our remaining ‘unknown’ causes of death may be classified in the future as drug-use complications or overdoses. Sadly, it is also likely that the numbers of deaths has continued to increase in this cohort. This analysis highlights the grave impacts the overdose crisis is having on some of the most vulnerable populations of PLWH in the province, greatly thwarting our public health efforts to improve the survival and quality of life for all PLWH in BC.

While Treatment as Prevention efforts, publicly-funded ART, and free-of-charge HIV-care have been tools that have led to great declines in morbidity and mortality among PLWH, persistent barriers to care remain [2, 38]. Our study demonstrates that HIV-related mortality was the second leading cause of death in our cohort of PLWH in BC. The observation of elevated rates of HIV/AIDS-related mortality at a time when ART carried few side-effects, had high tolerability, and was highly efficacious, signals important gaps in the provision of HIV-related care for under-served populations [39]. The LISA cohort in particular has a high proportion of women and people who use drugs; communities that experience unique barriers to ART and healthcare [40, 41]. Efforts to optimize engagement of under-served communities and support them through culturally appropriate, low-barrier, integrated primary care that considers mental health and substance use, is critical to improve health outcomes overall. Evidence suggests that integrated care focused on HIV and substance use issues in community settings improve retention and adherence to ART [42].

There are several limitations in this analysis to consider, including the end of follow-up being in June 2017. Notably, the increase in deaths attributed to overdose is one of the key reasons for missingness in vital statistics data, and reliably we could only look back until June 2017. Moreover, cause of death may be subject to misclassification bias leading to over-estimates of HIV-related causes of death. While we had a relatively small sample size for assessing mortality, it is striking that more than 1 in every 5 people enrolled in our study had died within the first 10 years of the first LISA survey being administered. The survey administered at entry in to the LISA cohort did not allow for longitudinal assessment of key social characteristics and certain health care engagement variables, including persistent barriers to accessing care. Finally, LISA is a non-probability sample which was not representative geographically or demographically of PLWH enrolled in the DTP. However, that this study over-sampled women and people who used drugs allowed for an assessment of health care utilization and health outcomes of under-served PLWH in the province.

Despite the limitations considered, there are several strengths to this project including the linkage of survey data to an ongoing clinical registry. Our study also makes clear that taking a siloed approach to treating HIV and supporting PLWH is no longer sufficient in populations of PLWH where substance use and barriers to care are common and comorbidities are the rule, not the exception. Integrated health service delivery models that offer low-barrier primary and preventative care and an opportunity to engage PLWH in care for substance use disorders and other chronic and infectious conditions is needed now more than ever. Supporting the health and longevity of underserved PLWH will thus require innovative clinical care strategies that acknowledge structural barriers and prioritize integrated care for HIV and related health concerns.

## Conclusions

Overall, these findings suggest that underserved PLWH experience a significant burden of mortality related to overdoses; high rates of HIV/AIDS-related mortality despite ART being universally provided; and age-related chronic comorbidities that are leading to premature deaths. In the BC setting where HIV care and support, including ARV, is offered free of charge, nearly 1 in 5 deaths in our sample were due to HIV-related complications, calling attention to the unique and persistent barriers to care some PLWH experience. The majority of deaths in our study were attributed to overdoses or drug-related complications. The overdose crisis in BC suggests that this will continue to be a leading cause of death among PLWH, undermining public health efforts in BC to address health inequities and support longevity and health among underserved PLWH in the province. Modern therapeutics and public health efforts, including Treatment as Prevention, have allowed us to imagine the “End of AIDS” [43], but to ignore emerging public health crises, including the overdose epidemic, would be doing a disservice to PLWH. While BC has often been at the forefront of harm reduction strategies [44], these finding highlights the dire need for life-saving interventions, including increased safe supply options, that prioritize the needs of people who use drugs. This work underscores the need for a syndemics approach to tackling intersecting public health threats, and for low-barrier, integrated care services with special focus on preventative health care, infectious diseases, mental health and addiction.

## Abbreviations

AIC: Akaike Information Criterion
AIDS: Acquired Immunodeficiency Syndrome
AOR: Adjusted odds ratio
ART: Antiretroviral therapy
BC: British Columbia
BC-CfE: British Columbia Centre for Excellence in HIV/AIDS
COPD: Chronic Obstructive Pulmonary Disease
CAD: Canadian dollar
DTP: Drug Treatment Program
HCV: Hepatitis C
HIV: Human Immunodeficiency Virus
IDU: Injection drug use
IQR: Interquartile range
LISA: Longitudinal Investigation into Supportive and Ancillary health services Study
NADM: Non-AIDS-defining malignancies
PLWH: People living with HIV
PY: Person years
RR: Rate ratio
